# Effects of Substituting Fish Meal by Chlorella Meal in Practical Diet on Growth, Feed Utilization, and Flesh Quality of Pacific White Shrimp (*Penaeus vannamei*)

**DOI:** 10.1155/2024/9969518

**Published:** 2024-06-19

**Authors:** Menglu Li, Xiaoqin Li, Wenxiang Yao, Yuanyuan Wang, Lufan Li, Xiangjun Leng

**Affiliations:** ^1^ National Demonstration Center for Experimental Fisheries Science Education Shanghai Ocean University, Shanghai, China; ^2^ Centre for Research on Environmental Ecology and Fish Nutrition (CREEFN) of the Ministry of Agriculture Shanghai Ocean University, Shanghai, China; ^3^ Shanghai Collaborative Innovation for Aquatic Animal Genetics and Breeding Shanghai Ocean University, Shanghai, China

## Abstract

This study investigated the effects of substituting fish meal (FM) in practical diet by chlorella meal on the growth, feed utilization, and flesh quality of Pacific white shrimp (*Penaeus vannamei*). First, a basal diet was prepared with 200 g/kg FM inclusion (FM-20), and then chlorella meal was used to reduce FM inclusion to 150 g/kg (FM-15), 100 g/kg (FM-10), 50 g/kg (FM-5), and 0 g/kg (FM-0), corresponding to the replacement levels of 25%, 50%, 75%, and 100% of dietary FM, respectively. Shrimp (1.37 ± 0.10 g) were fed with the five isonitrogenous and isolipidic diets for 56 days. No significant difference was observed in feed conversion ratio (FCR) and weight gain (WG) between FM-20 and FM-15 group (*P* > 0.05), but when chlorella meal substituted 50% of dietary FM, WG, protein and lipid retention, and n-3/n-6 PUFAs in flesh were significantly reduced with significant increase in FCR (*P* < 0.05). Survival, feed intake, meat yield, apparent digestibility coefficient of crude protein, dry matter, and flesh shear force showed no significant difference between FM-20 and substituted groups (*P* > 0.05). When dietary FM was totally substituted by chlorella meal, the body yellowness and redness and essential amino acid content in flesh, including Lys and Met, were significantly reduced (*P* < 0.05). No significant differences were found in flesh total collagen, crude lipid, crude protein, serum biochemical indexes, flesh texture profiles (hardness, springiness, etc.), water holding capacity, antioxidant capacity, fatty acid, free amino acid composition, and muscle fiber density among the five treatments (*P* > 0.05). To sum up, in a practical diet with 200 g/kg FM inclusion, chlorella meal successfully replaced 25% of dietary FM without adverse impacts on the growth and feed utilization, and the substitution of 75% of dietary FM did not negatively affect the flesh quality of Pacific white shrimp.

## 1. Introduction

Pacific white shrimp, *Penaeus vannamei*, is the major shrimp species cultured in the world, and the global production was about 5.8 million tons in 2020 [1]. Generally, the commercial feed for the shrimp contains relatively high level of fish meal (FM). Due to the limited resource and the increasing price of FM, to develop sustainable protein sources is urgent to reduce the inclusion level of FM in aquatic feeds, especially in shrimp feeds.

Microalgae have been regarded as a promising alternative that can replace fish meal and ensure sustainability standards in aquaculture [2], and *Chlorella* and *Spirulina* could be major algae protein source in farmed fish nutrition [3]. Chlorella is the general name of the genus *Chlorella* in Chlorophyta, and it is the first artificially cultured microalgae. Chlorella contains plenty of nutrients such as polysaccharides, amino acids, unsaturated fatty acids, cytochromes, vitamins, and some unknown growth factors [4]. Previous studies have shown that chlorella meal could partially or completely replace dietary FM for narrow clawed crayfish (*Pontastacus leptodactylus*) [5], largemouth bass (*Micropterus salmoides*) [6], rainbow trout (*Oncorhynchus mykiss*) [7], zebrafish (*Danio rerio*) [8], Pacific white shrimp [9], and crucian carp (*Carassius auratus*) [10]. In addition, chlorella meal was also used to substitute vegetable proteins such as soybean meal in Pacific white shrimp diet without affecting the growth [11].

The demand for protein of animals is essentially the demand for essential amino acids. When the composition and quantity of essential amino acids in diet meet the requirements, animals would have a good growth performance. Conversely, protein efficiency and protein retention will be reduced, negatively affecting the health and even causing pathological symptoms, such as intestinal deformation [12] and cataracts [13]. Exogenous supplementation of limiting amino acids is an effective nutritional strategy to balance the amino acid composition in diet. In tiger shrimp (*Penaeus monodon*) [14], American lobster (*Homarus americanus*) [15], and kuruma shrimp (*Marsupenaeus japonicus*) [16], the supplementation of limiting amino acids in diets promoted the growth performance. Thus, it should be considered to supplement limiting essential amino acids in low FM diet to balance the amino acid composition.

Cholesterol is a kind of sterol lipid rich in animal somatic cells and blood. Cholesterol is also an essential nutrient for crustaceans [17], which plays important roles in the survival, growth, development, molting, reproduction, osmotic pressure regulation, and resistance against environment stress. Most crustaceans do not have the ability to synthesize cholesterol from scratch using acetyl-CoA as a precursor, so they must obtain exogenous cholesterol from food. Generally, animal protein sources such as FM and chicken meal have high cholesterol content, while plant protein sources and algae contain little cholesterol. When the feeds lacked cholesterol, kuruma shrimp [18], Pacific white shrimp [19], and giant freshwater prawn (*Macrobrachium rosenbergii*) [20] presented significantly lower survival and growth.

Our previous study has shown that chlorella meal successfully substituted 40% of dietary FM (336 g/kg) without significant effects on the growth performance and flesh quality of Pacific white shrimp [21]. In that study, the FM inclusion in control diet was high up to 560 g/kg with relatively single protein source, and the amino acid balance was not considered. Generally, commercial shrimp diets contain 150–250 g/kg of FM and a variety of non-FM protein sources. In practical diets, how much FM could be substituted by chlorella meal, and how about the impacts on flesh quality? Thus, in the present study, chlorella meal was used to replace FM with the addition of cholesterol and amino acids, to evaluate the impacts on growth, feed utilization, and flesh quality of white shrimp. The results will guide the use of chlorella meal in shrimp diets.

## 2. Materials and Methods

### 2.1. Experimental Diets and Design

First, a basal diet was prepared with 200 g/kg FM inclusion (FM-20). Then, four isolipidic (60.0 g/kg) and isoproteic (420.0 g/kg) diets were made by decreasing FM level to 150, 100, 50, and 0 g/kg with the inclusion of chlorella meal (FM-15, FM-10, FM-5, and FM-0), respectively. In the 4 FM-substituted diets, microencapsulated histidine, methionine, and cholesterol were added to reach the same levels as the FM-20l group. Y_2_O_3_ was added (0.5 g/kg) as indicator to determine nutrients digestibility. All ingredients were crashed, then screened through 80-mesh sieve. Sinking pellets (1.2 mm diameter) were made by a single screw extruder (Longxiang Food Machinery Factory, LX-75, Hebei, China) with pelleting temperature of 86 ± 4°C. After the postcooking (95°C, 20 min) and air-drying, the pellets were sealed (4°C) until use.

*Chlorella sorokiniana* meal was supplied by Demeter Bio-Tech Co., Ltd. (Zhuhai, China), which contained 607.9 g/kg crude protein, 100.3 g/kg crude lipid, 54.2 g/kg crude ash, 240.3 g/kg carbohydrate, and 60.6 g/kg moisture. The preparation of microencapsulated amino acids referred to the description of Chen et al. [22]. Briefly, the amino acid and corn starch mixture (1 : 1) was gelatinized for 30 min (90°C) and then dried (60°C) and ground. Diet formula and proximate composition are shown in Table 1, and amino acid and fatty acid composition is presented in Tables 2 and 3.

### 2.2. Experimental Animal and Feeding Management

Pacific white shrimp with an age of 15 days were provided by Shanghai Lianwang Aquatic Technology Co., Ltd. (Shanghai, China). All shrimp were fed commercial diet (60 g/kg crude lipid and 400 g/kg crude protein) at Binhai Aquaculture Station (Shanghai, China) for 1 month. Then, 1,000 healthy shrimp (1.37 ± 0.10) g were randomly allotted to 20 cages (1.0 m × 1.0 m × 1.2 m) with 50 shrimp per cage and four cages per treatment. Cages were hung in indoor pools (5.0 m × 3.0 m × 1.5 m) with 10 cages per pool. Shrimp were fed the five diets for 56 days with daily feeding intake ranging from 8% (the early period) to 4% (the late period) of body weight. The feeding frequency was four times a day (6 : 45, 11 : 45, 16 : 45, and 10 : 45). The daily feed intake was adjusted based on weather conditions and feeding behavior to ensure that shrimp ate up diets within 2 hr after feeding. Every 5 days, the feces was siphoned out, and about one-fourth water was renewed with filtered pond water. The water was detected daily, and the temperature, pH, dissolved oxygen, salinity, nitrite, and ammonia nitrogen were as follows: 22−30°C, 7.6–8.7, ≥5.0 mg/L, 0.4–0.8‰, ≤0.15, and ≤0.2 mg/L.

### 2.3. Sampling

At the beginning of trial, 20 shrimp were sampled and stored at −20°C for the initial analysis of proximate composition. After 56 days of feeding, all shrimp were deprived of diets for 24 hr, then the number was counted, and the weight was measured for individual cage to calculate growth performance such as feed conversion ratio (FCR), weight gain (WG), and survival. Five shrimp per cage were selected for body length and body weight measurement to determine condition factor (CF). The hemolymph of the five shrimp was collected from the pericardial cavity, centrifuged (4°C, 4,000 *r*/min, 10 min), and then the supernatant was kept at −80°C until use. The hepatopancreas was sampled and measured weight to estimate the hepatopancreas somatic index (HSI). After the peeling off, the muscle was collected and weighed for meat yield calculation [23] and then preserved (−80°C) to detect fatty acids, amino acids, collagen content, and biochemical indexes. Another three shrimp per cage were selected, and the second and the third abdominal segment was used to determine flesh texture and boiling (steaming) loss, respectively. In addition, some tail segments were stored in GD fixative for flesh histology observation, and some were stored at −20°C to measure thawing loss. In addition, three shrimp per cage were selected to evaluate body surface color after 5 min of boiling.

### 2.4. Measurements and Methods

#### 2.4.1. Growth and Body Indices

(1) $$\begin{array}{c} \text{WG }\left(\%\right)=100\times \left[\frac{\left(\text{final body weight }\left(\text{g}\right)-\text{ initial body weight }\left(\text{g}\right)\right)}{\text{initial body weight }\left(\text{g}\right)}\right]. \end{array}$$

(2) $$\begin{array}{c} \text{FCR}=\frac{\text{dry feed intake }\left(\text{g}\right)}{\left[\text{final body weight }\left(\text{g}\right)-\text{ initial body weight }\left(\text{g}\right)\right]}. \end{array}$$

(3) $$\begin{array}{c} \text{Survival }\left(\%\right)=100\times \left(\frac{\text{final shrimp number}}{\text{initial shrimp number}}\right). \end{array}$$

(4) $$\begin{array}{c} \text{FI }\left(\text{g}/\text{shrimp}\right)=\frac{\text{feed intake per cage }\left(\text{g}\right)}{\text{ final shrimp number}}. \end{array}$$

(5) $$\begin{array}{c} \text{Meat yield }\left(\%\right)=100\times \left[\frac{\text{whole muscle weight }\left(\text{g}\right)}{\text{ body weight }\left(\text{g}\right)}\right]. \end{array}$$

(6) $$\begin{array}{c} \text{HSI }\left(\%\right)=100\times \left[\frac{\text{hepatopancreas weight }\left(\text{g}\right)}{\text{body weight }\left(\text{g}\right)}\right]. \end{array}$$

(7) $$\begin{array}{c} \text{CF }\left(\text{g}/{\text{cm}}^{3}\right)=100\times \left[\frac{\text{body weight }\left(\text{g}\right)}{\text{body length }{\left(\text{cm}\right)}^{3}}\right]. \end{array}$$

#### 2.4.2. Proximate Composition of Diets, Shrimp, and Flesh

The contents of moisture, crude protein, crude lipid, and ash in diets, shrimp, and flesh were detected using AOAC method [24]. The moisture content was detected by drying samples to constant weight in oven (105°C). The ash content was measured by burning samples in muffle furnace (550°C, 6 hr). The crude lipid and crude protein contents were determined using Soxhlet extractor (Soxtec2050, Sweden) and Kjeldahl system method (FOSS Tecator, 2300 AutoAnalyzer Sweden).

#### 2.4.3. Nutrient Digestibility and Retention

The inductively coupled plasma atomic emission spectroscopy (HPLC-ICPMS, Vista MPX, Varian, California, USA) was used to determine the Y_2_O_3_ contents in feces and diets. Apparent digestibility coefficients of crude protein and dry matter (ADCP and ADDM) and lipid and protein retention ratio (LRR and PRR) were calculated as follows:

(8) $$\begin{array}{c} \text{ ADCP }\left(\%\right)\;=\;100\;\times \;\left[1\;-\;\left(\text{Dietary }Y_{2}O_{3}\;\times \text{ Faecal CP}\right)\;/\;\left(\text{Faecal }Y_{2}O_{3}\;\times \text{ Dietary }\mathrm{CP}\right)\right]. \end{array}$$

(9) $$\begin{array}{c} \text{ADDM }\left(\%\right)\;=\;100\;\times \;\left(1\;-\text{ Dietary }Y_{2}O_{3}\;/\text{ Faecal }Y_{2}O_{3}\right). \end{array}$$

(10) $$\begin{array}{c} \text{LRE }\left(\%\right)=100\times \frac{\text{ lipid gain }\left(\text{g}\right)}{\text{ lipid intake}\left(\text{g}\right)}. \end{array}$$

(11) $$\begin{array}{c} \text{PRE }\left(\%\right)=100\times \frac{\text{ protein gain }\left(\text{g}\right)}{\text{ protein intake }\left(\text{g}\right)}. \end{array}$$

#### 2.4.4. Amino Acid and Fatty Acid Profiles in Diets and Flesh

For the determination of amino acid composition, freeze-dried sample (20 mg flesh or 50 mg diet) was hydrolyzed with 6 M HCl in vacuum (24 hr, 110°C). After the drying, diluting, and filtering, the hydrolysate was used to detect amino acid contents with amino acid analyzer (S-433D, Sykam, Germany).

The free amino acid profile in flesh was determined referring to the description by Li et al. [21]. Briefly, wet sample (0.3 g) was homogenized (5% trichloroacetic acid), ultrasonicated, and centrifuged. Then, the supernatant was adjusted pH to 2.0 with 6 M NaOH. After filtering, the filtrate was used to measure free amino acid composition by amino acid analyzer (Waters ACQUITY Ultra High-Performance LC/MS, USA).

The measurements of fatty acid were conducted with the boron trifluoride method [21]. The extracted lipid was mixed with boron trifluoride methanol solution for water bath (100°C, 25 min), and then benzene and methanol solution was added for another water bath. Then, the sample was mixed with n-hexane and distilled water for centrifuging (10 min, 3,000 *r*/min). The supernatant was sampled for analyzing fatty acid profiles with gas chromatograph–mass spectrometer (Agilent Technologies 7890B, USA).

#### 2.4.5. Hemolymph and Flesh Biochemical Indices

Hemolymph samples were used to detect triglyceride (TG), total cholesterol (T-CHO), albumin (ALB), total protein (TP), and glucose (GLU), with GPO-PAP method, GPO-PAP method, bromocresol green method, Coomassie brilliant blue method, and oxidase method, respectively. The lactic acid (LA), malondialdehyde (MDA), total antioxidant capacity (T-AOC), lutathione peroxidase (GSH-Px), and superoxide dismutase (SOD) in flesh were determined with colorimetric method, TBA method, ABST method, and WST-1 method, respectively. All the measurements were performed with commercial kits produced by Nanjing Jiancheng Bioengineering Institute (Nanjing, China).

Alkaline hydrolysis method was used to measure the hydroxyproline (Hyp) content in flesh. Then, the collagen content was obtained by multiplying the Hyp content by eight (AOAC[25]). The heat-soluble collagen (HS) was measured referring to the description of Li et al. [21], and the content of heat-insoluble collagen (HIS) was calculated as follows:

(12) $$\begin{array}{c} \text{HIS}=\text{Total collagen content}-\text{HS}. \end{array}$$

#### 2.4.6. Body Color Parameters

The shrimp was cooked in boiling water for 5 min, and then the second abdominal segment was used for color analysis such as *a* ^*∗*^ (redness), *b* ^*∗*^ (yellowness), and *L* ^*∗*^ (lightness). The measurement was performed with WSC-S colorimeter (Shanghai Precision Scientific Instrument Co., Ltd., Shanghai, China)

#### 2.4.7. Flesh Texture and Water Holding Capacity

The shrimp was cooked for 5 min, then the shell was peeled off, and the flesh of the second abdominal segment was used to measure texture characteristics with texture analyzer (Tengba Instrument Technology Co., Ltd., Shanghai, China). Texture parameters, including chewiness, hardness, springiness, resilience, gumminess, and cohesiveness, were measured with cylindrical probe (25 mm × 25 mm), 40% deformation, test speed 1 mm/s, and contact sensing 5 gf. Shear force was measured using Warner–Bratzler shear cutter with 1 mm/s test speed and 5 gf contact sensing.

For water holding capacity measurement, the flesh from the third abdominal segment (W1) was cooked or steamed for 5 min and then weighed (W2). Another block of flesh (W1) was frozen for 24 hr (−20°C) and then thawed and weighed (W2). The water holding capacity was calculated as follows:

(13) $$\begin{array}{c} \text{Boiling (thawing, steaming) loss (\%)}=100\times \left[\frac{\left(\text{W1}\left(g\right)–\text{W2}\left(g\right)\right)}{\text{W1}\left(g\right)}\right]. \end{array}$$

#### 2.4.8. Flesh Histology

After the dehydrating in alcohol solutions and embedding in paraffin, flesh samples were cut into sections with 5 *μ*m thickness (Leikn RM2235 slicer, Germany), then stained with hematoxylin and eosin, and sealed with neutral gum. An imaging microscope (Nikon YS100, Japan) was used to observe the histological structures of muscle for muscle fiber counting and muscle fiber density calculation.

### 2.5. Statistical Analysis

All experimental data were expressed as mean ± standard deviation (SD) and then analyzed with statistical software (SPSS 26.0). One-way analysis of variance (ANOVA) was conducted to detect the significant differences (*P* < 0.05) between the observed responses. The statistical significance among groups was determined by Tukey's multiple range tests. In addition, orthogonal polynomial contrast was performed to determine the linear and/or quadratic effects.

## 3. Results

### 3.1. Growth Performance and Body Index

After 60 days of feeding, all groups presented high survival (>95%). Compared with the FM-20 group (control), the substitution of 25% FM by chlorella had no significant effect on FBW, WG, and FCR (*P* > 0.05), but the WG was significantly decreased, and FCR was increased (*P* < 0.05) when the substitution level reached 50%. The HSI of all substituted groups was significantly lower than that of the control group (*P* < 0.05). No significant differences in survival, FI, CF, and meat yield were detected among the five groups (*P* > 0.05) (Table 4).

### 3.2. Composition and Color Parameters of the Body

In Table 5, when chlorella meal completely substituted dietary FM (FM-0), the crude protein, and crude lipid contents, the yellowness and redness values were significantly decreased, while the moisture content was significantly increased (*P* < 0.05). The FM-15 group showed significantly higher body surface yellowness than the FM-20 group (*P* < 0.05). No significant difference in the crude ash content and lightness was observed among the five groups (*P* > 0.05).

### 3.3. Nutrient Utilization

In Table 6, LRR in the FM-10, FM-5, and FM-0 group and PRR and ADCP in the FM-5 and FM-0 group were significantly lower than those in the FM-20 group (*P* < 0.05). In addition, the FM-0 group showed significantly lower ADDM than the FM20 group (*P* < 0.05).

### 3.4. Flesh Composition

In Table 7, no significant differences in flesh moisture, crude lipid, crude protein, total collagen, and heat-insoluble collagen contents were detected among the five groups (*P* > 0.05). When chlorella meal substituted 150 g/kg FM (75%, FM-5 group), the crude ash content was significantly increased (*P* < 0.05).

### 3.5. Flesh Texture and Water Holding Capacity

In Table 8, dietary chlorella meal level showed linear and quadratic effects on flesh shear force (*P* < 0.05), which was significantly lower in the FM-0 and FM-5 groups than that in the FM-15 group (*P* < 0.05). No significant difference was detected in flesh hardness, springiness, chewiness, gumminess, cohesiveness, resilience, cooking loss, steaming loss, and thawing loss among the five groups (*P* > 0.05).

### 3.6. Serum and Flesh Biochemical Indices

Neither the contents of TP, T-CHO, TG, ALB, and GLU, in serum, nor the SOD, T-AOC, GPx activity, MDA, and LA contents in flesh were significantly affected by the replacement of FM with chlorella meal (*P* > 0.05) (Table 9).

### 3.7. Amino Acid Composition in Flesh

In Table 10, the complete FM replacement with chlorella meal (FM-0 group) significantly decreased the contents of essential amino acids, lysine, and methionine in flesh (*P* < 0.05). There were no significant differences in flesh contents of total amino acids and the other amino acids among the five groups (*P* > 0.05).

Seventeen free amino acids were tested in the flesh, and proline presented the highest content, followed by glycine and arginine. No significant difference was found in flesh contents of delicious amino acids (DAAs), total free amino acids (TFAAs), and 17 free amino acids among the five groups (*P* > 0.05) (Table 11).

### 3.8. Fatty Acid Composition in Flesh

Compared with the control group, the ratios of LOA, ALA, and n-6 PUFAs in the FM-0, FM-5, and FM-10 groups were significantly increased, while the ratios of EPA, DHA, and n-3 PUFAs in the FM-0 and FM-5 groups were decreased (*P* < 0.05). When chlorella meal substituted 50% of dietary FM, the ratio of n-3/n-6 PUFAs was significantly reduced (*P* < 0.05). No significant difference was observed in the ratios of SFAs, MUFAs, ARA, and DPA in flesh among the five groups (*P* > 0.05) (Table 12).

### 3.9. Flesh Histology

**Figure 1:**
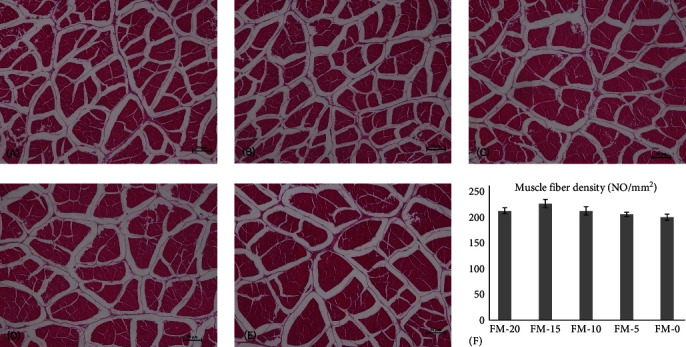
Effects of substituting fish meal with chlorella meal on muscle morphology of *P. vannamei*. (A, B, C, D, and E represent FM-20, FM-15, FM-10, FM-5, and FM-0 groups, respectively (200x); (F) statistical map of muscle fiber density).

In Figure 1, no significant difference in muscle fiber density was observed among the five groups (*P* > 0.05). The FM-15 group showed the highest muscle fiber density (*P* > 0.05).

## 4. Discussion

### 4.1. Growth Performance

In the present study, chlorella meal successfully substituted 25% of dietary FM without adverse effects on FCR and WG, which was similar to our previous result in white shrimp (40%) [21]. However, Pakravan et al. [9] reported that dietary fish meal (400 g/kg) could be completely replaced (100%) by chlorella with no adverse effects on shrimp performance. It is noting that the growth performance in that study was not good with low WG (<150%) and high FCR (1.85–2.42), which might affect the conclusion.

In aquatic feeds, some problems still exist in FM replacement by plant protein sources, including low palatability, amino acid imbalance, and low digestibility and utilization [26]. Chlorella is a natural food for white shrimp, and squid visceral meal was used as attractant; thus, no significant difference was found in feed intake in this study (Table 4). Therefore, the palatability of feed is not the main reason to limit the growth performance of shrimp fed high chlorella diet in the present study.

Compared with FM, chlorella meal contains less histidine and methionine without cholesterol. Li et al.[21] found that the histidine and methionine contents accounted for only 71.6% and 70.0% of the control diet, when chlorella meal completely substituted dietary FM (560 g/kg). In this study, microencapsulated histidine, methionine, and cholesterol were added to the FM-substituted diets to reach the same levels as the control diet (FM-20). When chlorella meal substituted 25% of dietary FM, no significant effects were observed on FCR and WG, while the higher replacement (≥50%) significantly decreased the growth performance. Xi et al. [6] also reported that the FCR was significantly increased when chlorella meal replaced 75% of FM in diet of largemouth bass. The present results showed that there are some other factors limiting the high inclusion of chlorella meal besides histidine, methionine, and cholesterol. For example, chlorella lacks bioactive substances such as taurine and hydroxyproline, which are rich in fish meal. In addition, the low digestibility of chlorella meal may be another reason. Xi et al. [6] reported that the digestibility of dry matter, protein, of chlorella meal was 72.94% and 84.38%, much lower than those of FM (81.29% and 92.62%). In the previous study [21] and in this study, the apparent digestibility of crude protein and dry matter was significantly reduced when high level of FM was substituted by chlorella meal, which may be connected with the thick cell wall of chlorella [2], affecting the nutrient digestibility. As the protein digestibility of chlorella meal was lower than that of fish meal, the available amino acid contents were further reduced in the high chlorella meal diets. Xu et al. [27] conducted transcriptome analysis to investigate the possible mechanism of chlorella replacing fish meal on growth and found that the high replacement of fish meal with chlorella negatively affected nutrients utilization by regulating the signaling pathways related to protein digestion and absorption and aggravated lipid catabolism of the body, thereby affecting the growth of shrimp.

### 4.2. Flesh Physical and Chemical Characteristics and Color Parameters

In a diet with 560 g/kg FM inclusion, the flesh shear force and hardness of white shrimp were significantly decreased when chlorella meal replaced 60% FM without supplementing amino acid [21]. However, in this study, there was no significant difference in flesh hardness and shear force among all the groups, indicating that dietary supplementation of cholesterol and essential amino acids eliminated the negative effects on flesh texture of shrimp. Wang et al. [28] and Jiang et al. [29] once reported that dietary supplementation of tryptophan and arginine increased the collagen and crude protein contents and promoted the flesh firmness of grass carp. The flesh firmness was affected by many factors such as muscle fiber diameter, cross-sectional area, and density. In brown trout (*Salmo trutta*) [30] and Atlantic salmon [31], flesh firmness was reported to be positively correlated with muscle fiber density and negatively correlated with muscle fiber diameter. In the present study, no significant difference was detected in muscle fiber density among different groups, which was consistent with the analysis of flesh hardness. In gilthead seabream (*Sparus aurata*), the decreasing muscle fiber diameter and the increasing muscle fiber density did not significantly affect flesh hardness [32], indicating that the flesh hardness is not only related to muscle fiber but also influenced by some other factors. In Atlantic halibut (*Hippoglossus hippoglossus* L.), the effect of collagen cross-linking degree and content played more roles in flesh hardness than muscle fiber density did [33]. As the main protein in connective tissue, the type, content, and structure of collagen affect the tenderness, hardness, and chewiness of flesh [34]. In collagen, heat-insoluble collagen plays an important role due to its high thermal stability. In this study, there was no significant difference in the contents of total collagen and heat-insoluble collagen among all the groups. The present study used a practical formula including several animal proteins such as fish meal, meat and bone meal, and squid visceral meal, which contained abundant hydroxyproline. In addition, several essential amino acids were added to balance the amino acid composition in the FM-substituted diets. Therefore, the substitution of FM by chlorella meal did not greatly affect the collagen synthesis.

After slaughter, the antioxidant capacity of muscle significantly affects flesh quality, and the oxidative damage can reduce the water holding capacity [35]. Oxidative damage is closely related to the antioxidant enzyme activities such as SOD, PO, and GPx [36], and the MDA content can reflect the oxidative damage degree of lipids and proteins caused by excessive reactive oxygen species [37]. In this experiment, T-AOC, MDA content, SOD, and GPx activities were not significantly affected by chlorella meal inclusion, indicating that appropriate amount of chlorella meal produced little effect on flesh antioxidant properties of *P. vannamei*. The content of collagen in flesh greatly influences flesh water holding capacity [38], and the connective tissue in muscle can prevent water evaporation and juice overflow through the dense membrane sheath[39]. Above the denaturation temperature, collagen fibers would contract to form a gel network with high water retention capacity [40]. In this experiment, the substitution of FM with chlorella meal presented no significant effect on collagen content and steaming, cooking, and thawing loss of flesh.

The shrimp body color of depends on the content of carotenoids in external tissue, especially in the epidermal layer. Astaxanthin is the predominant carotenoid in shrimp, and crustacean cannot synthesize astaxanthin by themselves; thus, the addition of carotenoids to the diet can increase the pigmentation of shrimp [41]. Xi et al. [6] once reported that the replacement of 75% of dietary FM with chlorella significantly increased the yellowness and redness of the dorsal muscle of largemouth bass, as chlorella contains abundant carotenoids. Shrimp can convert lutein from chlorella meal to form astaxanthin, but the amount of astaxanthin obtained from this conversion is obviously lower than that obtained directly from FM. Thus, it is easy to understand the significant decrease in *a* ^*∗*^ and *b* ^*∗*^ values of body surface in the FM-0 group.

### 4.3. Flesh Nutrition and Flavor

The dietary inclusion of chlorella meal did not change the contents of crude lipid, crude protein, and moisture in the flesh of gilthead seabream [42] and in the body of *P. vannamei* [9]. The similar results were also observed in this study, but the lysine and methionine contents in flesh were significantly decreased in the FM-0 group, which may be due to the decrease of lysine level in diet and the low utilization of the supplemented methionine.

The type and content of free amino acids give different flavors to the flesh. Generally, aspartic acid, glycine, glutamic acid, and alanine have a fresh taste, while glycine, threonine, alanine, and serine present sweetness [43]. No significant difference in flavor amino acid content of flesh was found among all the groups, indicating that the taste of shrimp flesh was not significantly affected by dietary chlorella meal.

Studies have shown that n-3 fatty acids give the flesh of turbot (*Psetta maxima*) an aromatic smell [44], while n-6 fatty acids give the tench (*Tinca tinca* L.) flesh an unpleasant smell [45]. Most of the animals cannot synthesize n-6 and n-3 PUFAs de novo, which can be obtained from feeds or from precursors [46]. In this study, with the increasing proportion of chlorella meal replacing fish meal, the C18 : 2 and C18 : 3 ratio was increased, and the C20 : 5, C22 : 6 ratio was decreased, which was consistent with the previous results in high FM diets [21]. Pakravan et al.[9] reported that dietary ARA, DHA, and EPA contents reduced, while the contents in flesh of *P. vannamei* increased with the increasing level of chlorella replacing fish meal. In rainbow trout, the total substitution of FM by chlorella meal significant increased serum LOA, ALA, and EPA level [7]. The replacement of 30% of dietary FM by chlorella meal significantly increased the n-6 PUFAs levels, but did not affect the n-3 PUFAs levels of gilthead seabream [42]. The different results may be related to the amount and type of chlorella meal and the diet composition.

## 5. Conclusion

In a practical diet with 200 g/kg FM inclusion, chlorella meal successfully replaced 25% of dietary FM (50 g/kg) without affecting the growth performance of Pacific white shrimp. The complete substitution of dietary FM with chlorella meal significantly decreased the whole body crude protein content, flesh essential amino acid content, and body surface yellowness and redness but did not affect flesh textural characteristics, water holding capacity, and free amino acid and collagen contents. The proportion of FM replaced by chlorella meal is 25% (50 g/kg) according to response of growth and flesh quality.

## Figures and Tables

**Table 1:** Ingredients and proximate composition of diets (air dry basis, g/kg).

Ingredients^(1)^	FM-20	FM-15	FM-10	FM-5	FM-0
Fishmeal	200.0	150.0	100.0	50.0	0.0
Chlorella meal	0.0	56.6	113.3	169.9	226.6
Wheat flour	284.5	277.3	269.8	262.6	254.9
Soybean meal	150.0	150.0	150.0	150.0	150.0
Soybean oil	10.0	9.0	8.0	7.1	6.1
Vitamin and mineral premix^(2)^	10.0	10.0	10.0	10.0	10.0
Ca (H_2_PO_4_)_2_	20.0	20.0	20.0	20.0	20.0
Other ingredients^(3)^	325.5	325.5	325.5	325.5	325.5
Coated histidine (50%)	0.0	0.6	1.4	2.0	2.8
Coated methionine (50%)	0.0	0.7	1.4	2.1	3.0
Cholesterol	0.0	0.28	0.56	0.84	1.12
Total	1,000.0	1,000.0	1,000.0	1,000.0	1,000.0
Proximate composition					
Crude protein	418.8	416.8	417.1	416.1	417.7
Crude lipid	61.8	59.1	60.2	59.5	59.3
Crude ash	77.7	70.7	64.1	67.3	72.3
Moisture	71.5	70.6	71.2	72.3	73.5

*Note*: ^(1)^The ingredients were purchased from the Yuehai Feed Company (Zhejiang, China), and the protein contents of the ingredients are as follows: TASA fish meal 682.1 g/kg, wheat flour 145.5 g/kg, and soybean meal 436.7 g/kg. ^(2)^One kilogram of vitamin and mineral premix contained vitamin A 10,000,000 IU, vitamin D3 2,500,000 IU, vitamin E 1,00,000 IU, vitamin K3 8,600 mg, vitamin B1 11,760 mg, vitamin B2 20,000 mg, vitamin B6 19,600 mg, vitamin B 1,250 mg, D-biotin 200 mg, folic acid 3,800 mg, niacinamide 59,700 mg, d-pantothenic acid 44100 mg, potassium 200.00 g, magnesium 58.200 g, cuprum 3.550 g, zinc 5.400 g, manganese 1.950 g, cobalt 0.198 g, selenium 0.040 g, iodine 0.248 g, and moisture ≤6%. ^(3)^Other ingredients (g/kg) contained soy protein concentrate, 50.0; corn gluten meal, 60.0; meat and bone meal, 50.0; wheat gluten, 50.0; squid visceral meal, 50.0; brewers dried yeast, 50.0; soybean lecithin, 15.0; and Y_2_O_3_, 0.5.

**Table 2:** Amino acid composition of diets, fish meal, and chlorella meal (g/kg, dry matter).

Parameters	FM-20	FM-15	FM-10	FM-5	FM-0	FM	Chlorella meal
Essential amino acid							
Ile	17.0	17.5	17.7	17.5	17.6	27.5	20.2
Leu	33.7	33.9	33.5	34.2	34.2	52.6	43.4
Thr	17.8	17.3	17.3	17.3	17.6	28.7	22.7
Phe	24.8	24.7	25.5	26.6	25.2	35.9	21.8
Lys	23.8	23.2	22.9	22.5	22.4	52.1	31.5
His	12.6	11.9	12.2	11.7	12.2	20.7	7.7
Arg	24.8	24.4	25.3	24.4	24.5	40.9	28.5
Val	21.5	21.5	21.6	21.4	21.6	33.7	25.5
Met	10.2	10.0	10.5	9.7	9.8	20.3	7.1
Nonessential amino acid							
Asp	37.9	37.0	37.7	37.2	37.9	61.0	42.8
Glu	63.9	61.7	63.4	61.6	62.2	87.5	55.8
Gly	22.3	22.3	22.1	22.1	22.6	41.3	27.9
Ala	24.0	25.2	25.2	24.9	26.6	44.2	42.5
Cys	3.7	3.6	3.7	3.7	3.6	4.1	2.5
Tyr	17.8	17.5	18.4	17.7	16.9	22.9	17.1
Ser	20.4	20.2	20.4	21.0	23.0	26.1	18.9
Pro	21.6	21.6	21.2	22.0	21.3	28.4	32.5
Total amino acids	397.9	393.7	398.6	395.5	399.2	627.9	448.3

**Table 3:** Fatty acid composition of diets (percentage of total fatty acids, %).

Parameters	FM-20	FM-15	FM-10	FM-5	FM-0
C14 : 0	1.10	0.95	0.83	0.78	0.57
C15 : 0	0.14	0.13	0.14	0.14	0.13
C16 : 0	12.04	12.42	13.70	14.63	14.99
C17 : 0	0.31	0.34	0.37	0.25	0.39
C18 : 0	3.17	3.36	3.59	3.31	3.38
C20 : 0	0.16	0.17	0.17	0.16	0.17
C24 : 0	0.14	0.13	0.13	0.13	0.13
SFAs	17.05	17.48	18.93	19.39	19.66
C16 : 1	2.60	2.45	2.38	2.46	2.37
C18 : 1	22.98	23.81	22.70	22.02	22.84
C20 : 1	1.81	1.71	1.69	1.69	1.70
C24 : 1	0.39	0.38	0.42	0.40	0.41
MUFAs	27.78	28.34	27.19	26.57	27.32
C18 : 2	32.18	33.43	34.18	34.87	34.87
C20 : 4	1.09	0.90	0.84	0.90	0.89
n-6 PUFAs	33.28	34.33	35.02	35.77	35.77
C18 : 3	3.24	4.75	6.13	7.40	8.34
C20 : 5	7.53	6.13	5.05	4.53	3.49
C22 : 5	0.94	0.77	0.72	0.28	0.30
C22 : 6	10.17	8.18	6.97	6.05	5.12
n-3 PUFAs	21.89	19.84	18.87	18.27	17.25

SFAs, saturated fatty acids; MUFAs, monounsaturated saturated fatty acids; PUFAs, polyunsaturated fatty acids.

**Table 4:** Effects of substituting FM with chlorella meal on growth performance and body index of *P. vannamei*.

Parameters	Diets					Pr > *F*		
	FM-20	FM-15	FM-10	FM-5	FM-0	ANOVA	Linear	Quadratic
IBW (g)	1.37 ± 0.10	1.37 ± 0.08	1.37 ± 0.06	1.37 ± 0.09	1.37 ± 0.10	—	—	—
FBW (g)	15.67 ± 0.18^a^	15.23 ± 0.18^a^	14.09 ± 0.35^b^	13.69 ± 0.29^b^	13.64 ± 0.36^b^	0.001	0.001	0.069
WG (%)	1043.8 ± 13.4^a^	1011.7 ± 13.4^a^	928.7 ± 25.8^b^	899.5 ± 21.4^b^	895.6 ± 26.4^b^	0.004	0.001	0.346
Survival (%)	95.50 ± 2.51	95.50 ± 3.41	97.52 ± 1.89	98.00 ± 2.30	96.00 ± 4.32	0.663	0.475	0.342
FI (g/shrimp)	20.09 ± 0.17	20.11 ± 0.32	19.90 ± 0.16	20.07 ± 0.17	20.06 ± 0.28	0.710	0.786	0.466
FCR	1.43 ± 0.01^a^	1.49 ± 0.02^a^	1.58 ± 0.05^ab^	1.64 ± 0.05^b^	1.66 ± 0.07^b^	0.001	0.001	0.069
CF (g /cm^3^)	1.14 ± 0.05	1.10 ± 0.05	1.13 ± 0.05	1.14 ± 0.06	1.14 ± 0.05	0.444	0.541	0.304
HSI (%)	4.74 ± 0.40^a^	4.31 ± 0.39^b^	4.34 ± 0.30^b^	4.30 ± 0.37^b^	4.38 ± 0.42^b^	0.003	0.012	0.005
Meat yield (%)	54.15 ± 1.50	54.64 ± 2.38	53.72 ± 2.52	54.14 ± 1.94	54.86 ± 1.87	0.194	0.308	0.195

IBW, initial body weight; FI, feed intake; FBW, final body weight; WG, weight gain; FCR, feed conversion ratio; CF, condition factor; HSI, hepatopancreas somatic index. Pr > *F*, significant probability associated with *F*-statistic. *Note*. In the same row, values with different superscripts mean significant difference (*P* < 0.05).

**Table 5:** Effects of substituting FM with chlorella meal on body composition and color parameters of *P. vannamei* (wet weight, g/kg).

Parameters	Diets					Pr > *F*		
	FM-20	FM-15	FM-10	FM-5	FM-0	ANOVA	Linear	Quadratic
Moisture	746.8 ± 5.1^b^	743.7 ± 3.3^b^	737.3 ± 5.5^b^	749.9 ± 3.8^b^	787.9 ± 19.2^a^	0.001	0.001	0.001
Crude protein	193.1 ± 3.9^a^	194.9 ± 4.5^a^	197.3 ± 6.0^a^	191.7 ± 3.6^a^	160.0 ± 14.2^b^	0.001	0.001	0.001
Crude lipid	24.82 ± 1.04^a^	24.11 ± 2.01^a^	23.91 ± 2.46^a^	22.08 ± 1.23^ab^	19.02 ± 1.46^b^	0.002	0.001	0.069
Crude ash	26.75 ± 1.70	26.69 ± 1.92	25.80 ± 1.87	26.71 ± 1.04	25.51 ± 0.99	0.688	0.324	0.876
Lightness (*L* ^*∗*^)	57.95 ± 2.22	57.93 ± 2.15	58.42 ± 2.37	59.24 ± 1.91	58.33 ± 2.83	0.500	0.261	0.502
Redness (*a* ^*∗*^)	30.81 ± 2.83^ab^	32.49 ± 2.70^a^	30.29 ± 2.51^ab^	28.73 ± 1.15^b^	25.09 ± 2.68^c^	0.001	0.001	0.001
Yellowness (*b* ^*∗*^)	37.52 ± 2.37^b^	40.73 ± 2.09^a^	37.06 ± 2.72^b^	37.43 ± 1.44^b^	34.71 ± 1.43^c^	0.001	0.001	0.001

L ^*∗*^, a ^*∗*^, and b ^*∗*^ represent lightness, redness, and yellowness, respectively. *Note*. In the same row, values with different superscripts mean significant difference (*P* < 0.05).

**Table 6:** Effects of substituting FM with chlorella meal on nutrient utilization of *P. vannamei* (%).

Parameters	Diets					Pr > *F*		
	FM-20	FM-15	FM-10	FM-5	FM-0	ANOVA	Linear	Quadratic
PRR	33.22 ± 0.40^a^	32.48 ± 0.35^a^	31.04 ± 1.05^ab^	29.04 ± 0.83^b^	23.52 ± 0.93^c^	0.001	0.001	0.001
LRR	29.99 ± 0.34^a^	29.34 ± 0.31^a^	27.03 ± 0.89^b^	24.26 ± 0.66^c^	20.64 ± 0.79^d^	0.001	0.001	0.005
ADDM	55.11 ± 1.05^a^	54.41 ± 1.20^ab^	53.74 ± 3.38^ab^	51.68 ± 0.92^ab^	50.43 ± 0.48^b^	0.041	0.003	0.523
ADCP	78.79 ± 0.75^a^	77.89 ± 1.56^a^	76.86 ± 1.14^a^	72.40 ± 0.73^b^	71.01 ± 0.31^b^	0.001	0.001	0.045

PRR, protein retention ratio; LRR, lipid retention ratio; ADDM, apparent digestibility coefficients of dry matter; ADCP, apparent digestibility coefficients of crude protein. *Note*. In the same row, values with different superscripts mean significant difference (*P* < 0.05).

**Table 7:** Effects of substituting FM with chlorella meal on flesh composition of *P. vannamei* (wet weight, g/kg).

Parameters	Diets					Pr > *F*		
	FM-20	FM-15	FM-10	FM-5	FM-0	ANOVA	Linear	Quadratic
Moisture	743.42 ± 5.65	746.08 ± 6.75	748.71 ± 6.50	750.44 ± 2.71	743.47 ± 4.38	0.300	0.610	0.065
Crude protein	221.45 ± 0.90	222.29 ± 6.09	219.30 ± 0.57	219.00 ± 4.94	221.99 ± 2.87	0.733	0.754	0.410
Crude lipid	11.93 ± 0.87	11.64 ± 0.83	11.84 ± 0.55	11.83 ± 0.38	11.17 ± 0.52	0.523	0.228	0.441
Crude ash	10.56 ± 0.35^b^	10.93 ± 0.11^ab^	10.77 ± 0.18^ab^	11.24 ± 0.23^a^	11.23 ± 0.03^a^	0.010	0.002	0.776
Total collagen	3.62 ± 0.13	3.65 ± 0.22	3.63 ± 0.22	3.61 ± 0.17	3.63 ± 0.26	0.997	0.987	0.923
HS collagen	2.02 ± 0.13	2.01 ± 0.15	2.01 ± 0.17	2.02 ± 0.17	2.06 ± 0.12	0.956	0.583	0.589
HIS collagen	1.62 ± 0.12	1.65 ± 0.12	1.60 ± 0.09	1.58 ± 0.04	1.57 ± 0.10	0.968	0.531	0.862

HS collagen, heat soluble collagen; HIS collagen, heat insoluble collagen. *Note*. In the same row, values with different superscripts mean significant difference (*P* < 0.05).

**Table 8:** Effects of substituting FM with chlorella meal on flesh texture characteristics and water holding capacity of *P. vannamei*.

Parameters	Diets					Pr > *F*		
	FM-20	FM-15	FM-10	FM-5	FM-0	ANOVA	Linear	Quadratic
Hardness (gf)	402.5 ± 31.3	405.8 ± 34.1	427.7 ± 20.8	418.8 ± 30.0	405.3 ± 27.9	0.416	0.577	0.113
Springiness	0.70 ± 0.023	0.71 ± 0.025	0.69 ± 0.016	0.70 ± 0.018	0.71 ± 0.015	0.395	0.614	0.638
Chewiness (gf)	201.48 ± 13.14	208.41 ± 10.61	211.81 ± 10.96	210.23 ± 11.93	206.23 ± 13.48	0.503	0.409	0.110
Gumminess (gf)	286.4 ± 19.2	291.8 ± 27.5	291.8 ± 27.5	303.6 ± 21.1	291.9 ± 27.2	0.419	0.379	0.116
Cohesiveness (gf)	0.71 ± 0.012	0.72 ± 0.026	0.72 ± 0.010	0.73 ± 0.019	0.71 ± 0.014	0.440	0.411	0.170
Resilience	0.61 ± 0.025	0.65 ± 0.043	0.64 ± 0.025	0.65 ± 0.034	0.63 ± 0.023	0.092	0.413	0.025
Shear force (gf)	2165.5 ± 142.5^ab^	2400.0 ± 178.0^a^	2174.5 ± 173.7^ab^	2117.8 ± 143.9^b^	1963.4 ± 173.6^b^	0.001	0.001	0.009
Steaming loss (%)	27.25 ± 1.63	28.66 ± 1.37	28.84 ± 2.43	28.02 ± 2.04	28.63 ± 2.12	0.477	0.336	0.323
Cooking loss (%)	29.68 ± 2.40	30.67 ± 1.84	30.34 ± 2.88	30.82 ± 2.42	30.07 ± 1.25	0.961	0.729	0.402
Thawing loss (%)	3.24 ± 0.29	3.29 ± 0.2	3.30 ± 0.23	3.10 ± 0.27	3.28 ± 0.14	0.664	0.758	0.935

*Note*. In the same row, values with different superscripts mean significant difference (*P* < 0.05).

**Table 9:** Effects of substituting FM with chlorella meal on serum and flesh biochemical indices of *P. vannamei*.

Parameters	Diets					Pr > F		
	FM-20	FM-15	FM-10	FM-5	FM-0	ANOVA	Linear	Quadratic
Serum								
TP (g/L)	71.26 ± 4.51	70.90 ± 3.86	70.32 ± 2.65	71.32 ± 4.40	70.48 ± 3.33	0.989	0.815	0.919
TG (mmol/L)	0.48 ± 0.04	0.48 ± 0.03	0.48 ± 0.04	0.48 ± 0.04	0.49 ± 0.03	0.995	0.784	0.798
T-CHO (mmol/L)	0.72 ± 0.07	0.72 ± 0.06	0.73 ± 0.07	0.73 ± 0.05	0.73 ± 0.06	0.999	0.941	0.958
GLU (mmol/L)	0.83 ± 0.06	0.83 ± 0.07	0.81 ± 0.04	0.83 ± 0.06	0.85 ± 0.06	0.843	0.635	0.323
ALB (g/L)	6.64 ± 0.39	6.68 ± 0.41	6.82 ± 0.67	6.65 ± 0.59	6.73 ± 0.25	0.966	0.817	0.773
Muscle								
SOD (U/mg prot)	6.87 ± 0.46	6.90 ± 0.13	6.81 ± 0.27	6.69 ± 0.31	6.75 ± 0.11	0.826	0.294	0.977
T-AOC (*μ*mol/g prot)	53.24 ± 4.62	53.43 ± 4.50	52.96 ± 3.08	53.00 ± 2.74	52.91 ± 3.62	0.999	0.803	0.995
GPx (*μ*mol/min/g prot)	2.37 ± 0.20	2.36 ± 0.22	2.35 ± 0.29	2.35 ± 0.22	2.31 ± 0.19	0.993	0.650	0.898
MDA (nmol/g prot)	402.6 ± 42.7	401.8 ± 49.0	398.7 ± 45.6	399.9 ± 41.1	386.7 ± 40.1	0.959	0.510	0.733
LA (mmmol/g prot)	0.49 ± 0.03	0.50 ± 0.05	0.51 ± 0.03	0.49 ± 0.06	0.51 ± 0.02	0.790	0.642	0.947

TP, total protein; TG, triglyceride; T-CHO, total cholesterol; GLU, glucose; ALB, albumin; SOD, superoxide dismutase; T-AOC, total antioxidant capacity; GSH-PX, glutathione peroxidase; MDA, malondialdehyde; LA, lactic acid.

**Table 10:** Effects of substituting FM with chlorella meal on flesh amino acid composition of *P. vannamei* (dry matter basis, g/kg).

Parameters	Diets					Pr > *F*		
	FM-20	FM-15	FM-10	FM-5	FM-0	ANOVA	Linear	Quadratic
EAAs								
Thr	30.4 ± 2.2	30.4 ± 0.4	31.3 ± 0.4	31.0 ± 0.6	30.1 ± 0.4	0.722	0.968	0.271
Phe	38.3 ± 0.4	38.1 ± 0.6	38.6 ± 1.5	37.9 ± 1.0	37.7 ± 0.3	0.757	0.353	0.547
Lys	70.9 ± 0.7^a^	68.3 ± 0.8^ab^	67.8 ± 2.6^ab^	67.3 ± 1.7^ab^	66.9 ± 0.7^b^	0.031	0.004	0.182
His	17.7 ± 0.4	17.4 ± 0.5	17.4 ± 0.2	17.1 ± 1.1	17.2 ± 0.4	0.761	0.260	0.686
Arg	69.3 ± 2.3	69.3 ± 2.9	70.1 ± 0.3	69.9 ± 2.8	68.8 ± 1.1	0.960	0.909	0.534
Val	31.7 ± 0.5	31.3 ± 0.1	31.9 ± 0.3	32.0 ± 1.3	31.3 ± 0.8	0.762	0.989	0.601
Met	14.2 ± 1.4^a^	14.5 ± 1.0^a^	14.7 ± 1.5^a^	15.1 ± 1.6^a^	7.0 ± 1.4^b^	0.001	0.001	0.001
Ile	35.5 ± 1.1	36.8 ± 1.0	36.2 ± 0.6	36.5 ± 1.9	35.8 ± 1.4	0.777	0.936	0.348
Leu	67.8 ± 1.0	67.2 ± 1.3	678.±0.1	66.5 ± 0.2	66.4 ± 0.6	0.186	0.040	0.689
NEAAs								
Tyr	34.4 ± 0.4	35.0 ± 1.0	33.9 ± 0.9	33.7 ± 1.8	33.1 ± 0.5	0.350	0.090	0.557
Asp	81.9 ± 0.9	81.6 ± 0.9	81.3 ± 0.1	81.5 ± 0.7	81.4 ± 0.6	0.894	0.439	0.579
Pro	55.3 ± 0.6	54.8 ± 1.5	56.3 ± 0.9	56.0 ± 3.4	56.3 ± 1.7	0.866	0.409	0.994
Ser	30.7 ± 0.1	30.5 ± 0.5	31.0 ± 0.1	30.9 ± 0.9	30.6 ± 0.5	0.850	0.945	0.588
Glu	127.5 ± 3.5	124.5 ± 0.5	124.4 ± 3.2	124.2 ± 2.0	122.8 ± 0.1	0.210	0.040	0.551
Gly	56.4 ± 2.4	57.0 ± 2.5	56.6 ± 0.2	55.5 ± 0.9	55.5 ± 0.8	0.766	0.319	0.629
Ala	48.3 ± 2.3	49.8 ± 2.1	50.3 ± 0.1	49.7 ± 2.6	49.5 ± 0.9	0.799	0.540	0.329
Cys	5.3 ± 1.56	5.5 ± 0.55	5.4 ± 0.13	5.5 ± 0.65	5.3 ± 0.51	0.994	0.935	0.720
TEAAs	375.9 ± 4.4^a^	373.1 ± 4.1^a^	375.7 ± 2.1^a^	373.2 ± 5.4^a^	361.0 ± 2.0^b^	0.008	0.003	0.030
TAAs	815.7 ± 9.8	811.9 ± 7.7	814.9 ± 1.4	810.0 ± 9.1	795.6 ± 2.7	0.053	0.012	0.119

EAAs, essential amino acids; NEAAs, nonessential amino acids; TEAAs, totai amino acids; TAAs, total amino acids. *Note*. In the same row, values with different superscripts mean significant difference (*P* < 0.05).

**Table 11:** Effects of substituting FM with chlorella meal on flesh free amino acid composition of *P. vannamei* (wet weight, mg/100 g).

Parameters	Diets					Pr > *F*		
	FM-20	FM-15	FM-10	FM-5	FM-0	ANOVA	Linear	Quadratic
Asp ^*∗*^	2.54 ± 0.10	2.48 ± 0.15	2.55 ± 0.20	2.54 ± 0.14	2.58 ± 0.19	0.949	0.622	0.708
Thr	149.7 ± 4.3	147.2 ± 4.4	149.4 ± 6.1	149.0 ± 2.3	147.2 ± 8.4	0.963	0.763	0.921
Ser	17.33 ± 1.11	17.49 ± 0.40	17.70 ± 0.65	17.20 ± 0.44	17.33 ± 0.36	0.898	0.810	0.600
Glu ^*∗*^	44.52 ± 2.80	46.67 ± 2.31	45.94 ± 2.50	46.25 ± 2.18	44.43 ± 3.07	0.755	0.901	0.245
Gly ^*∗*^	673.9 ± 25.7	682.9 ± 9.0	676.4 ± 32.0	675.5 ± 29.6	679.2 ± 52.6	0.997	0.958	0.945
Ala ^*∗*^	239.3 ± 16.6	233.2 ± 11.3	235.6 ± 4.6	241.7 ± 11.1	242.3 ± 8.7	0.822	0.490	0.492
Cys	5.99 ± 0.77	5.87 ± 0.45	6.24 ± 0.49	5.90 ± 0.49	6.03 ± 0.63	0.937	0.927	0.871
Val	22.92 ± 1.20	22.92 ± 0.99	22.36 ± 0.65	22.94 ± 0.82	22.38 ± 0.95	0.857	0.547	0.993
Met	6.58 ± 0.29	6.52 ± 0.15	6.49 ± 0.24	6.47 ± 0.41	6.45 ± 0.40	0.989	0.617	0.903
Ile	7.72 ± 0.83	7.94 ± 0.71	7.52 ± 0.79	7.73 ± 0.93	7.97 ± 0.23	0.940	0.838	0.674
Leu	21.99 ± 0.15	21.84 ± 0.96	21.55 ± 1.44	20.99 ± 1.70	20.41 ± 0.61	0.443	0.078	0.648
Tyr	16.38 ± 1.33	16.37 ± 0.92	16.55 ± 1.15	16.56 ± 0.79	16.03 ± 0.64	0.964	0.789	0.586
Phe	12.60 ± 0.82	12.31 ± 0.79	12.23 ± 0.66	11.81 ± 1.33	12.07 ± 1.37	0.910	0.430	0.737
Lys	93.57 ± 2.22	91.31 ± 6.87	89.28 ± 2.91	88.73 ± 6.40	88.10 ± 2.34	0.608	0.140	0.645
His	29.03 ± 0.84	30.09 ± 0.56	29.30 ± 1.22	28.88 ± 0.93	27.68 ± 1.20	0.122	0.053	0.079
Arg	601.4 ± 53.9	620.5 ± 56.5	590.5 ± 27.5	598.6 ± 41.2	591.3 ± 12.8	0.901	0.593	0.873
Pro	879.1 ± 20.4	841.6 ± 41.2	837.8 ± 16.5	854.3 ± 56.8	831.7 ± 48.5	0.630	0.286	0.575
DAAs	960.3 ± 40.7	965.2 ± 8.1	960.5 ± 29.2	966.0 ± 27.2	968.6 ± 59.1	0.998	0.802	0.948
TAAs	2824.5 ± 88.5	2807.3 ± 19.8	2767.4 ± 31.1	2795.1 ± 76.3	2763.2 ± 66.1	0.717	0.263	0.782

DAA, delicious amino acids ( ^*∗*^); TAAs, total amino acids.

**Table 12:** Effects of substituting FM with chlorella meal on flesh fatty acid composition of *P. vannamei* (percentage of total fatty acids, %).

Parameters	Diets					Pr > *F*		
	FM-20	FM-15	FM-10	FM-5	FM-0	ANOVA	Linear	Quadratic
C14 : 0	0.09 ± 0.01	0.09 ± 0.01	0.09 ± 0.01	0.09 ± 0.01	0.09 ± 0.01	0.962	0.795	0.826
C15 : 0	0.15 ± 0.01	0.15 ± 0.1	0.15 ± 0.01	0.15 ± 0.01	0.15 ± 0.01	0.962	0.999	0.661
C16 : 0	13.13 ± 0.35	13.16 ± 0.24	13.31 ± 0.21	13.38 ± 0.23	13.15 ± 0.30	0.710	0.604	0.315
C17 : 0	0.87 ± 0.01	0.88 ± 0.02	0.89 ± 0.01	0.87 ± 0.01	0.88 ± 0.02	0.410	0.999	0.371
C18 : 0	8.36 ± 0.06	8.25 ± 0.14	8.26 ± 0.08	8.26 ± 0.32	8.33 ± 0.20	0.926	0.931	0.409
C20 : 0	0.38 ± 0.03	0.39 ± 0.02	0.37 ± 0.00	0.37 ± 0.03	0.36 ± 0.01	0.559	0.134	0.832
SFAs	22.99 ± 0.41	22.92 ± 0.18	23.08 ± 0.15	23.13 ± 0.09	22.97 ± 0.20	0.806	0.699	0.573
C16 : 1	0.64 ± 0.01	0.64 ± 0.01	0.64 ± 0.02	0.64 ± 0.01	0.64 ± 0.06	0.998	0.899	0.914
C18 : 1 OLA	15.31 ± 0.42	14.79 ± 0.52	14.54 ± 0.09	14.49 ± 0.46	14.92 ± 0.25	0.141	0.158	0.030
C20 : 1	0.86 ± 0.02	0.86 ± 0.01	0.85 ± 0.02	0.86 ± 0.03	0.87 ± 0.03	0.895	0.637	0.690
MUFAs	16.81 ± 0.45	16.29 ± 0.50	16.03 ± 0.09	16.00 ± 0.45	16.43 ± 0.25	0.132	0.160	0.027
C18 : 2 LOA	13.13 ± 0.65^c^	14.11 ± 0.50^bc^	15.00 ± 0.22^b^	17.80 ± 0.28^a^	18.67 ± 0.30^a^	0.001	0.001	0.094
C20 : 2	2.14 ± 0.12	2.16 ± 0.06	2.21 ± 0.08	2.13 ± 0.04	2.13 ± 0.02	0.660	0.712	0.338
C20 : 4 ARA	2.83 ± 0.04	2.83 ± 0.10	2.74 ± 0.16	2.72 ± 0.08	2.70 ± 0.08	0.338	0.060	0.865
n-6 PUFAs	18.11 ± 0.76^c^	19.10 ± 0.43^bc^	19.95 ± 0.34^b^	22.64 ± 0.30^a^	23.51 ± 0.34^a^	0.001	0.001	0.148
C18 : 3 ALA	0.61 ± 0.04^d^	0.83 ± 0.04^cd^	1.09 ± 0.03^c^	1.74 ± 0.12^b^	2.33 ± 0.26^a^	0.001	0.001	0.003
C20 : 5 EPA	21.27 ± 0.59^a^	21.21 ± 0.20^a^	21.05 ± 0.42^a^	19.01 ± 0.72^b^	17.32 ± 0.10^c^	0.001	0.001	0.001
C22 : 5 DPA	0.99 ± 0.01	1.00 ± 0.09	0.96 ± 0.03	0.94 ± 0.03	0.94 ± 0.01	0.408	0.081	0.835
C22 : 6 DHA	19.22 ± 0.50^a^	18.66 ± 0.70^ab^	17.85 ± 0.13^bc^	16.53 ± 0.19^c^	16.51 ± 0.68^c^	0.001	0.001	0.598
n-3 PUFAs	42.09 ± 0.96^a^	41.69 ± 0.93^a^	40.94 ± 0.26^a^	38.22 ± 0.68^b^	37.10 ± 0.36^b^	0.001	0.001	0.048
PUFAs	60.40 ± 0.46^a^	60.81 ± 0.53^a^	60.85 ± 0.43^a^	58.11 ± 5.00^ab^	53.00 ± 4.68^b^	0.012	0.003	0.030
n-3/n-6 PUFAs	2.33 ± 0.15^a^	2.18 ± 0.09^ab^	2.05 ± 0.05^b^	1.69 ± 0.05^c^	1.58 ± 0.03^c^	0.001	0.001	0.409

SFAs, saturated fatty acids; MUFAs, monounsaturated fatty acids; PUFAs, polyunsaturated fatty acids. *Note*. In the same row, values with different superscripts mean significant difference (*P* < 0.05).

## Data Availability

All data generated or analyzed during this study are included in this article.
